# A Pioneer in Women's Health, Dr. James Marion Sims (1813-1883)

**DOI:** 10.7759/cureus.74733

**Published:** 2024-11-29

**Authors:** Heena K Vaswani, Shilpa Kshirsagar, Sukesh Kathpalia, Aishwarya Denge

**Affiliations:** 1 Obstetrics and Gynaecology, Dr. D Y Patil Medical College, Hospital and Research Centre, Dr. D Y Patil Vidyapeeth (Deemed to be University), Pune, IND

**Keywords:** first cancer hospital, first women hospital, sims position, sims speculum, vesicovaginal fistula, women health

## Abstract

James Marion Sims, one of the most well-known and respected surgeons in America, lived from January 25, 1813, to November 13, 1883. He was chosen to be the American Medical Association's president in 1876 and was amongst the first American doctors to gain recognition in Europe. He founded New York's first hospital exclusively for women, despite strong opposition. The creation of a surgical method for treating vesicovaginal fistulas, a serious side effect of difficult childbirth, is his most well-known accomplishment. In addition, he is credited with creating the Sims sigmoid catheter, Sims speculum, and Sims posture.

It would be difficult to find a more controversial figure in the history of medicine. In Bryant Park in New York City, a statue honoring him - the first one honoring a physician in the country - was constructed in 1894. It was taken down in 2018.

The primary sources of information on Sims and his life and work are reports on his medical experiments that he published in abundance and his own 471-page autobiography (which was condensed into a speech shortly before his death).

## Introduction and background

Childhood, education, and early career

The son of John and Mahala (Mackey) Sims, James Marion Sims - who liked to be called "Marion" - was born in South Carolina. He grew up in Lancaster Village, which is north of Hanging Rock Creek, where his father ran a shop, for the first twelve years of his life. Sims was sent to the newly founded Franklin Academy in Lancaster in 1825 after his father was elected sheriff of Lancaster County. Following a couple of years of study at South Carolina College, the forerunner of the University of South Carolina, Sims began working with Dr. Churchill Jones in Lancaster in 1832.

The forerunner to the Medical University of South Carolina, The Medical College of Charleston, offered a three-month course that he completed, but he felt it was too demanding. After relocating to Philadelphia, Pennsylvania, in 1834, he enrolled at the Jefferson Medical College, where he studied apathy and demonstrated little drive until graduating in 1835 with a medical degree.

He stated: "Apart from wanting to make a living, I had no special interest in my profession at first. I was genuinely ready at any time and at any moment to take up anything that offered, or that held out any temptation of money, because I realized that I could never make a fortune out of the practice of medicine."

He went back to practice in Lancaster. Till that particular day, "he had had no clinical experience, logged no actual hospital time, and had no experience diagnosing illnesses." Sims was dejected after the deaths of his first two patients, who were infants. After leaving, he opened a clinic in Mount Meigs, Alabama, which is close to Montgomery.

In 1836, Sims went to Lancaster to marry Theresa, the daughter of B.C. Jones and the niece of Churchill Jones; she had studied at the South Carolina Female Collegiate Institute. Sims and his spouse relocated to Cubahatchee, Alabama, in 1837, and they stayed there until 1840. From 1835 to 1837, he was in Mount Meigs.

After the couple relocated to Montgomery, Alabama, in 1840, they remained there until 1853 [1]. Theresa Jones had studied at the South Carolina Female Collegiate Institute [2]. Sims referred to this time as the "most memorable time" of his career [3]. In a matter of years, he "had the largest surgical practice in the State" and the largest practice of any physician in Montgomery at the time [1]. "He was immensely popular and greatly beloved" [1]. In 1877, he paid a visit back to the city and was received with hero status. 

Sims carried on mostly treating enslaved patients in Montgomery, who constituted two-thirds of the population, as a plantation physician [4]. For the women whose owners brought them to him for treatment, he constructed a hospital known as the Surgical Infirmary for Negroes. He started with four beds, but it became so popular that he constructed a second level, bringing the total number of beds to eight. According to one source, it was increased to twelve beds. "The first woman's hospital in history" is how some have referred to it. Additionally, it was the country's first hospital built exclusively for Black patients.

Gynecology was not a field in 1840, and neither Sims nor anybody else had any training in it. There were just books about midwifery. Gynecological illnesses, childbirth, and pregnancy were not studied by medical students. Medical students were frequently taught how to deliver infants on dummies. It wasn't until they started their practices that they saw their first clinical instances of women. "The practice of examining the female organs was considered repugnant by doctors." Sims also held a similar opinion; in his autobiography, he states: "If there was anything I hated, it was investigating the organs of the female pelvis" [4].

## Review

Medical career and achievements

A woman with a vesicovaginal fistula, a condition Sims had never seen previously, was brought to him in 1845. In the 1800s, vesicovaginal fistulas were considered "one of the most loathsome and disagreeable maladies to which females are subject," while not being fatal [5]. They were common, horrific, and socially devastating childbirth complications that impacted a lot of women [6]. There was no proven treatment or cure. Women who gave birth often had difficulties later since in those days; there was insufficient access to birth control and little understanding of the pregnancy and delivery procedures [7].

When a woman's bladder, cervix, and vagina get stuck between her pelvis and the fetal skull, it can cause vesicovaginal fistulas, which stop the flow of blood and cause tissue death. A hole is left when the necrotic tissue eventually sloughs off. Urine seeps from the vagina as it forms after this injury, resulting in incontinence. It is challenging to maintain cleanliness in the vagina because of the constant stream of urine that pours from it. Such problems with personal cleanliness frequently result in vaginal discomfort, scarring, and loss of vaginal function, in addition to social isolation. Similarly, Sims treated rectovaginal fistulas, a disorder in which the rectum tears the tissue separating it from the vagina, allowing gas and feces to escape and causing fecal incontinence [6].

**Figure 1:**
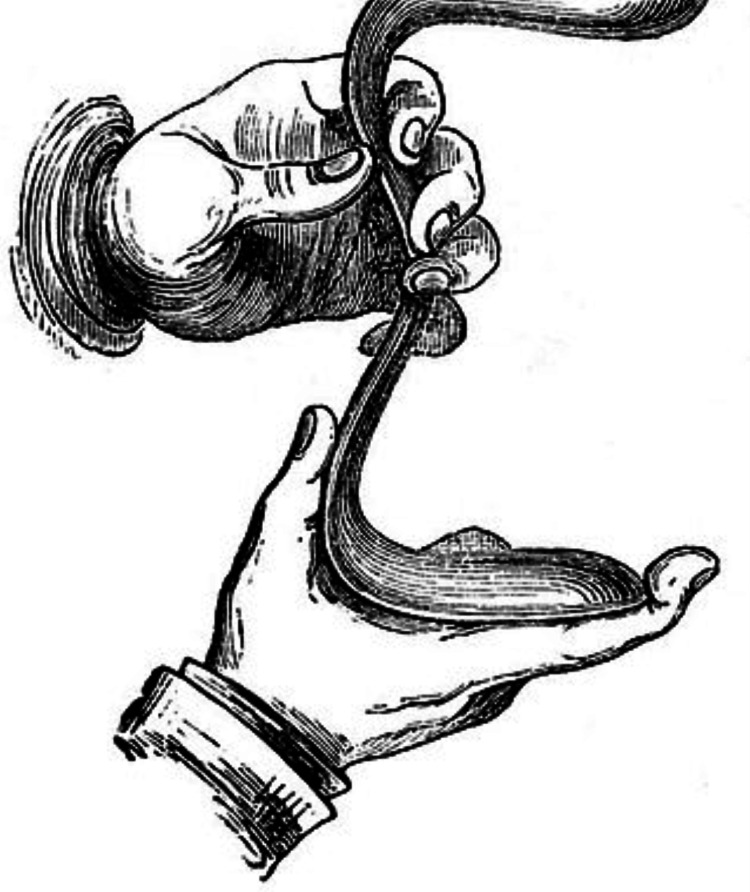
Sims' vaginal speculum Image credit: Wikimedia Commons [8]

Sims placed a woman in a knee-chest position and put his finger inside her vagina after seeing her for a pelvic injury sustained in a horse fall. Sims was able to see the vagina clearly as a result, which inspired him to look into fistula treatment [7]. He quickly created a device that was a forerunner to the present speculum (Figure 1) by arranging mirrors in strategic locations and utilizing a pewter spoon to make patient examinations easier. Consequently, he has been widely acknowledged as the creator of the instrument.

However, Dr. John Peter Mettauer and Dr. George Hayward had successfully surgically repaired vesicovaginal fistulas in Virginia in 1838 and Boston the following year, respectively; therefore, Sims was not the first person to do so [9]. Furthermore, the clinical work Medico-Chirurgical Observations (1676) by Henry van Roonhuyse described crucial remedial actions for these kinds of ailments that are still identifiable today.

Sims treated vaginal issues in enslaved Black women and girls between 1845 and 1849 by performing exploratory surgery on them. He doubled the capacity of his four-bed hospital by adding a second floor. He created methods that eventually became the foundation for contemporary vaginal surgery; silver wire, which he had a jeweler prepare, was a crucial element. As previously mentioned, Sims' vaginal speculum facilitated vaginal examination and surgery. He is also honored by the name of the rectal examination position, which involves the patient lying on their left side with their right knee bent against their abdomen and their left knee slightly bent.

Anesthesia

Sims employed no anesthetic during his treatments, despite the fact that it had recently been used in experiments. In 1868, Sims presented his research on nitrous oxide anesthesia, and in 1874, he published a paper on chloroform anesthesia [10]. Sims presented a paper at the New York Academy of Medicine in 1880 that was published shortly after regarding a death caused by anesthesia. In 1874, he addressed the New York State Medical Society on "The Discovery of Anaesthesia," asserting that Americans had discovered the practice prior to the British.

Because of the lack of understanding at the time regarding infection control and sanitation in operating rooms, Sims also made other mistakes. One patient, Lucy, almost passed away from sepsis when Sims operated on her without administering anesthesia, in front of a group of 12 doctors, and used a sponge in an experimental manner to remove urine from her bladder [7]. He carelessly let the sponge lodge in her bladder and urethra. Sims refused to use anesthesia when doing surgery, but he did often give the women opium afterward as this was a common therapeutic procedure at the time. But the main purpose of the opium was to make sure that these women who were being operated on wouldn't flee due to the unbearable pain.

Sims finally became an expert in his craft. One of his most important contributions was the invention of silver wire sutures in 1849, which eliminated the risk of lead poisoning or the infections linked to silk sutures, which Mettauer had used in 1838. In his surgical reports from 1852, Sims describes how he successfully repaired Anarcha's suture using silver wire. He then went on to fix fistulas in a number of additional African enslaved women.

**Figure 2:**
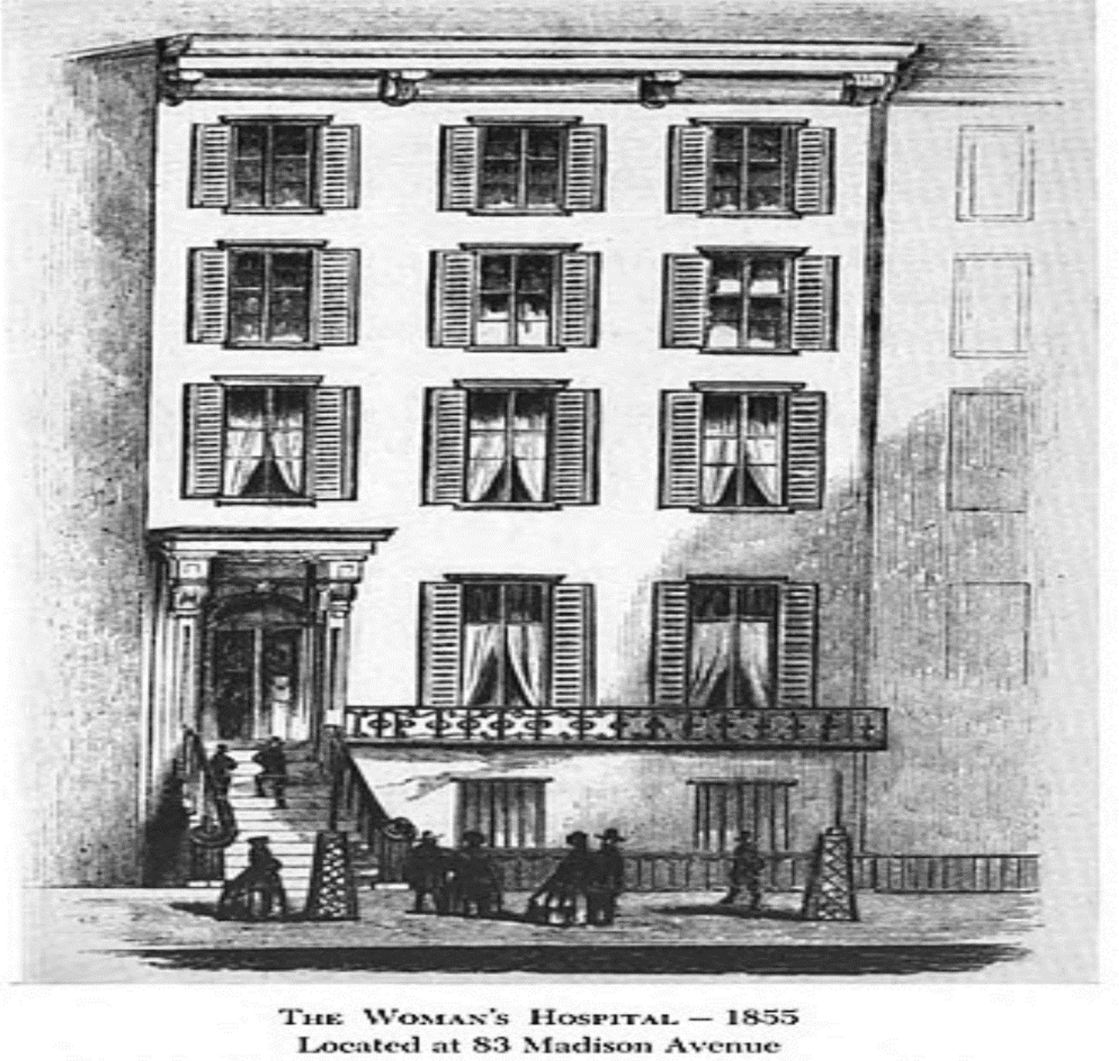
The Woman's Hospital Image credit: Courtesy of HathiTrust Digital Library [11]

**Figure 3:**
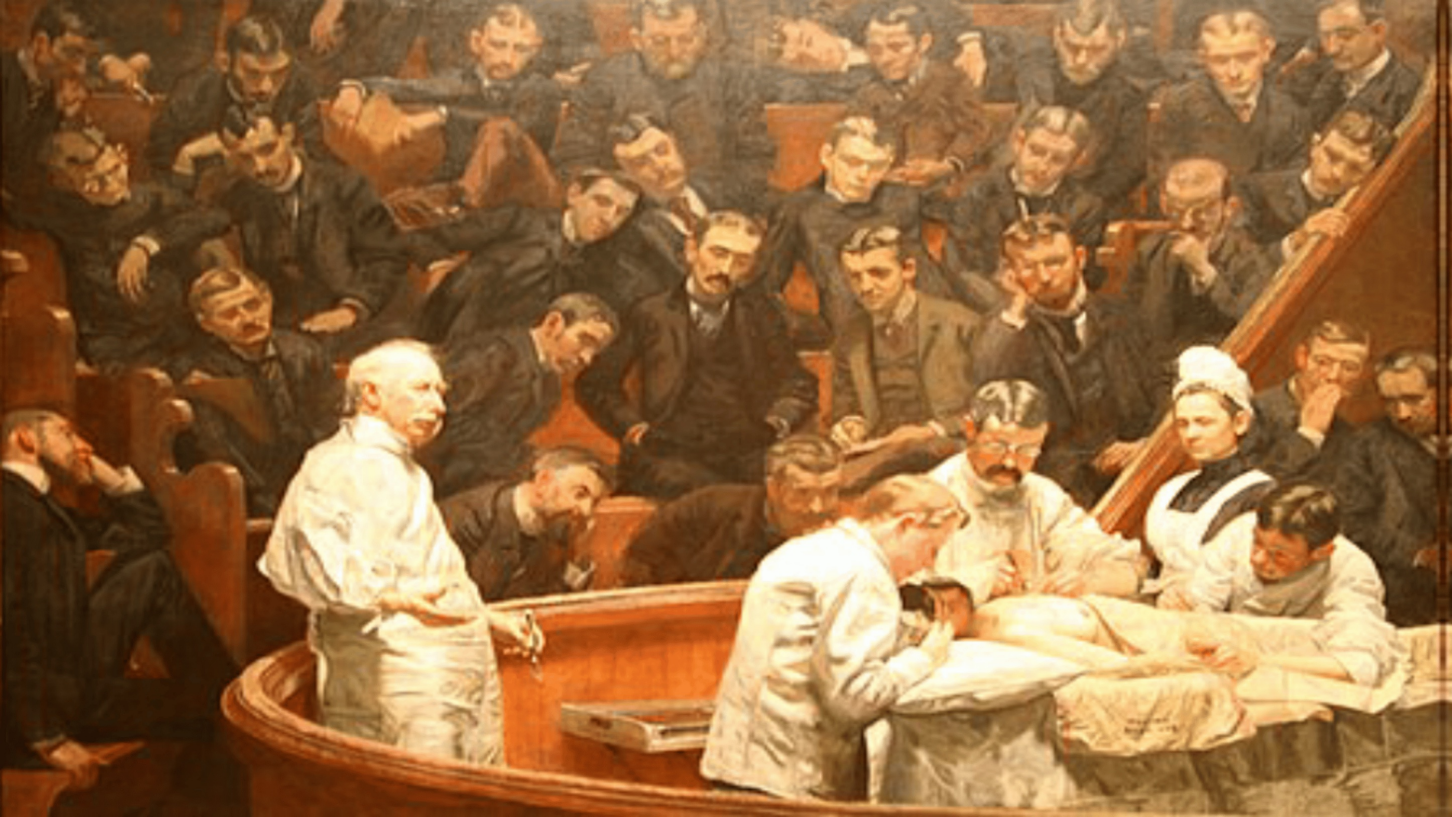
The first cancer hospital Image credit: Wikimedia Commons (Licensed under the Creative Commons Attribution 2.0 Generic license) [12]

He established the Woman's Hospital in 1855, which was the country's first hospital for women, except for his personal garden infirmary. When the Woman's Hospital first opened, its main goal was to help the underprivileged by performing Sims' procedure to repair vesicovaginal fistulas. There were no admitted "pay patients" (Figures 2-3). Situated near Sims' residence on Madison Avenue and 29th Street in a rented four-story house, the hospital's 30 beds were promptly occupied. At first, Sims performed alone, repairing one fistula every day without help from other medical professionals. Poor Irish immigrant ladies made up a large number of his patients.

Once again, Sims went back to New York in 1871 and worked at the Woman's Hospital, where he operated on women who had cancer. At the time, cancer was viewed as a disease that only affected the lower classes of society, and some people even believed it could be spread through sexual contact. Sims worked to change this perception, and the Woman's Hospital was eventually forced to ban cancer treatment after the powerful Ladies' Board successfully campaigned against it. Sims blasted the hospital's Board of Governors in 1874 during a meeting for refusing to treat cancer patients, even in the early stages of the disease. Sims played a key role in the founding of New York Cancer Hospital, America's first cancer institute, following a falling out with the board of the Woman's Hospital over the admittance of cancer patients. Sims was unanimously elected president of the American Medical Association in response to the care he got at the Woman's Hospital; he served in that capacity from 1876 to 1877 [1,2].

Contributions

The introduction of examining and surgical positioning, namely Sims' position, is significant. Instruments named after him include the Sims' sigmoid catheter and speculum. A campaign has been initiated to remove Sims' name from the instruments he invented, which include the Sims uterine curette, uterine sound, vaginal retractor, uterine scissors, and rectal speculum. The People of Colour (POC) committee will select new names.

Sims contributed to fertility treatment in the form of insemination and postcoital test. He was the first to perform surgeries like vaginal surgery for the correction of fistula. In the case of abdominal surgery, Sims urged a laparotomy to stem bleeding from gunshot wounds to this region, mend the damage, and drain the wound. When President James Garfield was shot in an attempt at his life, Sims responded via telegram from Paris, and his suggestions were eventually accepted. In the case of gallbladder surgery, in 1878, Sims removed stones from a distended gallbladder. He published the case believing it to be the first of its kind, and it was indeed the first of its kind.

With regard to cancer care, Sims pushed for the Woman's Hospital to admit cancer patients in spite of the prevalent notion that the disease was communicable.

Death

Sims experienced two episodes of angina in 1877 and a severe incidence of typhoid fever in 1880. Even though Sims experienced madness, W. Gill Wylie, one of his early 20th-century biographers, mentioned that he was constantly contriving instruments and conducting operations. Sims' convalescence took several months, during which he moved to Charleston to help with his recuperation. In June 1881, he made his way back to France. Sims started to complain of worsening heart problems when he returned to the United States in September 1881. He was in the middle of writing his autobiography and preparing to return to Europe when he passed away on November 13, 1883, in New York City (Manhattan), from a heart attack. He was interred at Green-Wood Cemetery in Brooklyn, New York.

## Conclusions

J. Marion Sims has been called the "father of gynecology" for his revolutionary approach to treating the diseases of women. He rose from humble origins to become a successful surgeon, teacher, and writer. His innovations included the first successful treatment for vesicovaginal fistula, the first gallbladder surgery, and the introduction of antiseptic principles in all areas of surgical treatment. The "Sims position" and "Sims speculum" are eponymic tributes to his accomplishments.

In recent years Sims has, however, become a focus of controversy because of his experimental surgeries on slave women. His powerful personality and messianic attitude led him to minimize moral problems and to bristle against opposition. Ethical principles of autonomy and beneficence are important criteria for evaluating Sims' research.
